# Real-World Use of Control-IQ Technology Is Associated with a Lower Rate of Severe Hypoglycemia and Diabetic Ketoacidosis Than Historical Data: Results of the Control-IQ Observational (CLIO) Prospective Study

**DOI:** 10.1089/dia.2023.0341

**Published:** 2024-01-05

**Authors:** Rishi Graham, Lars Mueller, Michelle Manning, Steph Habif, Laurel H. Messer, Jordan E. Pinsker, Eliah Aronoff-Spencer

**Affiliations:** ^1^ Division of Infectious Diseases and Global Public Health, Department of Medicine, University of California San Diego, La Jolla, California, USA.; ^2^ Tandem Diabetes Care, San Diego, California, USA.; Prior Publication: The study results were presented, in part, in abstract form at the 82nd ADA Scientific Session in June, 2022.

**Keywords:** Adverse events, Hypoglycemia, Diabetic ketoacidosis, Control-IQ technology, Type 1 diabetes

## Abstract

**Objective:**

Severe hypoglycemia (SH) and diabetic ketoacidosis (DKA) remain significant risks with intensive insulin therapy. While these adverse event (AE) rates are generally very low in advanced hybrid closed-loop (AHCL) clinical studies, prospectively collected real-world AE rates are lacking.

**Research Design and Methods:**

The Control-IQ Observational (CLIO) study was a single-arm, prospective, longitudinal, postmarket surveillance study of individuals with type 1 diabetes (T1D) age 6 years and older who began the use of t:slim X2 insulin pump with Control-IQ technology in the real-world outpatient setting. AEs were reported monthly over 12 months and were compared to historical data from the T1D Exchange. Patient-reported outcomes were assessed quarterly. All study visits were virtual.

**Results:**

Three thousand one hundred fifty-seven participants enrolled from August 2020 through March 2022. Two thousand nine hundred ninety-eight participants completed through 12 months. SH rates were significantly lower than historic rates for children (9.31 vs. 19.31 events/100 patient years, *d* = 0.29, *P* < 0.01) and adults (9.77 vs. 29.49 events/100 patient years, *d* = 0.53, *P* < 0.01). DKA rates were also significantly lower in both groups. Lower observed rates of AEs occurred independent of baseline hemoglobin A1c or prior insulin delivery method. Time in range 70–180 mg/dL was 70.1% (61.0–78.8) for adults, 61.2% (52.4–70.5) for age 6–13, 60.9% (50.1–71.8) for age 14–17, and 67.3% (57.4–76.9) overall. Reduction in diabetes burden was consistently reported.

**Conclusions:**

SH and DKA rates were lower for users of t:slim X2 with Control-IQ technology compared to historical data for both adults and children. Real-world use of this AHCL system proved safe and effective in this virtual study design. The study was registered at clinicaltrials.gov (NCT04503174)

## Introduction

The Diabetes Complications and Control Trial identified a threefold increase in severe hypoglycemia (SH) as the major risk associated with intensive insulin therapy.^1^ More recently, survey data from the type 1 diabetes (T1D) exchange showed 6% of individuals with T1D reported experiencing seizure or loss of consciousness due to hypoglycemia in the 3 months before questionnaire completion. At least one diabetic ketoacidosis (DKA) event requiring overnight hospitalization in the 3 months before the questionnaire was reported by 3% of participants. Insulin pump and continuous glucose monitoring (CGM) use was associated with a lower frequency of both SH and DKA.^2^

Prior randomized controlled trials (RCTs) of the t:slim X2 insulin pump with Control-IQ technology have shown significant improvements in time-in-range, with very low rates of SH and DKA across participants of different ages and baseline hemoglobin A1c (HbA1c) levels.^3–6^ A critique of these RCTs and similar studies of other devices is that study participants often receive extra supervision, and some studies required frequent visits to study centers, which may act as a barrier to enrollment that reduces diversity in the populations studied.^7^ As such, there is a lack of generalizable data on the rates of these acute diabetes complications with advanced hybrid closed-loop (AHCL) use outside of carefully supervised clinical trials.

Shortly after the release of Control-IQ technology in the United States, the COVID-19 pandemic produced a rapid virtualization of clinical trials and health care delivery globally.^8^ This transformation created opportunities for more accessible interventions to take advantage of technology that did not always require a clinic visit to use and operate, reducing the reliance on clinical encounters.

Consistent with this notion of relying on technology to facilitate clinical trial completion and advancing the concept of decentralized clinical trials,^9^ we report results from the Control-IQ Observational (CLIO) study. This was a single-arm, prospective, fully virtual longitudinal study of a large cohort of participants with T1D aged 6 years and older. The study was designed to assess the safety and effectiveness of the Tandem t:slim X2 insulin pump with Control-IQ technology in the real-world outpatient setting over 12 months of use. Enrollment and all monthly check-ins were performed through automated and virtual methods using a survey platform and remote device uploads. Adverse event (AE) rates include SH and DKA, glycemic outcomes, and patient-reported outcomes (PROs) with real-world use of this AHCL system are presented.

As the study was performed through multiple peaks of the COVID-19 pandemic, when in-person visits were at many times not possible, this study design offers potential insights into the feasibility of performing decentralized studies for postmarket follow-up.

## Research Design and Methods

The study was approved by the FDA under the Section 522 Postmarket Surveillance Studies Program. The Western IRB approved the study (IRB #1284748), and all participants signed informed consent before starting. The study was registered at clinicaltrials.gov and was performed in accordance with the principles of the Declaration of Helsinki. The authors assume responsibility for the accuracy and completeness of the data and analysis.

**FIG. 1.**
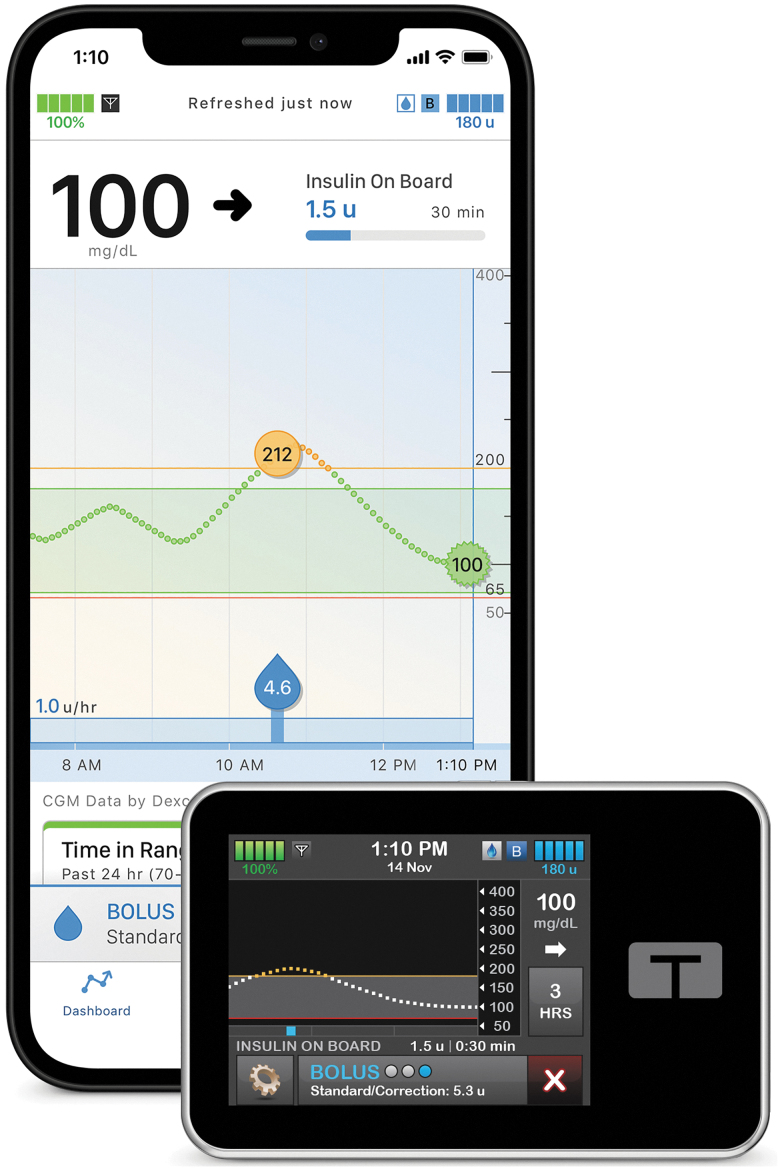
The Tandem t:slim X2 insulin pump with Control-IQ technology. The hybrid closed-loop system consists of (1) the Tandem t:slim X2 insulin pump, (2) the Control-IQ technology glycemic control algorithm embedded in the pump, (3) a compatible iCGM, and (4) an optional smartphone app (iOS or Android) that allows for bolusing from a personal phone, acts a secondary display for the pump, and uploads pump data automatically to the Tandem t:connect web application. iCGM, integrated continuous glucose monitor.

The CLIO study, unlike a typical postmarket device trial performed through enrollment at expert clinical sites, recruited individuals in the United States who were beginning use of the commercial t:slim X2 insulin pump with Control-IQ technology in the outpatient setting (Fig. 1). Potentially eligible individuals were invited to participate by email through their contact information registered in the Tandem Diabetes Care customer relationship management system, with a goal number of participants of different cohorts stratified by age (6–13, 14–17, 18+ years), prior HbA1c (≤8.5% [≤69 mmol/mol], >8.5% [>69 mmol/mol]), and prior therapy modality (prior pump or multiple daily injections [MDI] user, and prior CGM/non-CGM user). These specific cohorts were determined for regulatory approval. Enrollment occurred over a 19-month period from August 2020 through March 2022.

Key inclusion criteria included diagnosis of T1D, availability of a prestudy HbA1c measurement within the last 6 months, having been prescribed the t:slim X2 insulin pump with Control-IQ technology and agreed to use the system in closed-loop with the Dexcom G6 continuous glucose monitoring system for 12 months, age at least 6 years, using only lispro or aspart insulin, not pregnant or planning pregnancy in the next 12 months, reside full time in the United States, and agree to upload pump data at least every 3 months via personal computer or automatically using the t:connect mobile app. Key exclusion criteria included type 2 diabetes, age <6 years, use of any glucose-lowering therapy other than lispro or aspart insulin, or pregnancy.

Per study protocol, participants were also considered screen failures if pump data demonstrating use of Control-IQ technology had not been uploaded to the t:connect web application within 120 days of baseline survey completion.

Primary endpoints were safety: (1) the incidence rates of SH and DKA, and (2) the safety of the automatic population of CGM readings into the bolus calculator when Control-IQ technology was in use. Secondary endpoints were to assess (1) glycemic outcomes as a measure of efficacy of the system, and (2) patient-reported satisfaction with and trust in the system, diabetes impact, and sleep quality of Control-IQ technology users.

Participants first completed a baseline survey to obtain demographic data, which also included questions on current therapy modality, prior HbA1c level, insulin use, how Control-IQ technology was obtained, and concern about COVID-19 (Supplementary Material). Although not a planned comparison, baseline AE rates were also asked through the survey, but these events were not verified. There were no restrictions or guidance given on device settings or training beyond standard training materials and sessions offered in the real world, and individuals could perform virtual (remote) pump training if eligible as determined by their prescribing physician's pump start orders. Participants could also perform a remote software update of an existing Tandem pump to initiate Control-IQ technology use. Baseline and then monthly surveys to assess for AEs were performed through a website. There were no in-person study visits. Participants did their pump training and any follow-up with their regular providers independent of the study.

AEs to include SH and DKA was solicited through monthly surveys over the 12 months for each participant, with AE rates compared to historic data reported by Foster et al. (frequency of ≥1 AE of each type in the prior 3 months per participant).^2^ All calls to Tandem customer technical support were also screened for reportable AEs for enrolled participants. AEs on monthly surveys and those called in to technical support were verified with follow-up contact as per established postmarket follow-up procedures, with up to three outreach attempts to obtain more information and assistance with downloading data. AEs were then reviewed by the pricincipal investigator, who was able to use t:connect data and Tandem call center data to corroborate events. Independent review of the adjudicated AEs was performed by a physician at the University of California San Diego on quarterly basis to determine if there was any disagreement with the prior determinations.

Individual monthly self-reported AE counts were transformed into quarterly event rates to match the historic data.^2^ Events were binned into 90-day increments from the day the participant entered the study and counted as one if any event occurred within the 90-day period. To normalize, these individual patient event counts were annualized ( × 4/participant 90-day periods in study), then multiplied by 100 to provide outcomes per 100 patient years.

SH was defined as reportable in the protocol when assistance of another person was required to actively administer treatment due to altered mental status. DKA was defined as reportable in the protocol when the participant had (1) symptoms such as polyuria, polydipsia, nausea, or vomiting; and (2) treatment provided in a health care facility; and (3) diagnosis of DKA made by a health care provider and self-reported by the participant.

Similar to prior reports,^10^ participant outcomes were included if they met the minimum data requirement of 75% CGM data available for at least 21 days. Ninety-seven percent of participants who began the monthly surveys met this criterion.

### Glycemic outcomes

Glycemic results are presented overall and for adult (age 18+), adolescent (age 14–17), and pediatric (age 6–13) participants. Further subanalysis was performed by prior therapy modality to include MDI or prior pump therapy, and by baseline HbA1c.

### Patient-reported outcomes

PROs were collected at baseline, 3, 6, and 12 months and were chosen to assess satisfaction with and trust in the system, diabetes impact, and changes in sleep quality. The Diabetes Impact and Device Satisfaction Scale (DIDS) is a brief validated 11-item survey rated on a 10-point Likert scale, with two factors, including “device satisfaction” (7 items), and “diabetes impact” (4 items).^11^ The DAWN2 Impact of Diabetes Profile (DIDP) scale assessed the impact of diabetes on quality of life with seven items representing different dimensions of life (e.g., physical, financial, and so on), rated on a 7-point scale (1 = very positive impact, 7 = very negative impact), and scored as a composite.^12^ Quality of sleep was measured by a single item response to the question, “Thinking about the past month, how would you rate your sleep quality” (“Very Poor,” “Poor,” “Average,” “Good,” and “Very Good”).

### Statistical analysis

Data analysis as well as independent review of adjudicated AEs was performed by the University of California San Diego, Center for Health Design. The risk of AE (DKA, SH), represented as risk ratio per 100 patient years, was compared using a one-sided *t*-test (*α* = 0.05) to the expected values as extracted in an *a-priori* literature review. Effect size was defined as the absolute difference in risk ratios. The safety of automatically populating CGM readings into the bolus calculator was analyzed by calculating the number of times where consecutive CGM readings in the hypoglycemic range existed within 5 h after a correction bolus was given using an automatically populated CGM reading compared to when bolus recommendations were overridden. Secondary endpoints were calculated as means/medians as appropriate based on data extracted from the Tandem t:connect web application and PROs.

Sensor-glucose concentrations were analyzed per recent international consensus guidelines^13,14^ to include percent time in target range 70–180 mg/dL (3.9–10 mmol/L), time >180 mg/dL (>10.0 mmol/L), time >250 mg/dL (>13.9 mmol/L), time <70 mg/dL (<3.9 mmol/L), time <54 mg/dL (<3.0 mmol/L), and mean glucose. PRO scores were compared with Wilcoxon signed-rank hypothesis tests.

### Power analysis

An overall incidence rate of 25.35 events per 100 patient years was used in the power analysis for SH based on the *a-priori* literature review. We calculated that a sample size of 1354 completers would provide 80% power with a type 1 error rate (two-sided) of 5% to detect a difference, if there is one, between the expected SH rate and the proposed study sample SH event rate.

An overall incidence rate of 11.03 events per 100 patient years was used in the power analysis for DKA based on the *a-priori* literature review. We calculated that a sample size of 1282 completers would provide 80% power with a type 1 error rate (two-sided) of 5% to detect a difference, if there is one, between the expected DKA rate (11.03/100 PY) and the proposed study sample DKA event rate.

Additional details of the power analysis for each subgroup are provided in the Supplementary Material.

## Results

### Participant characteristics

**FIG. 2.**
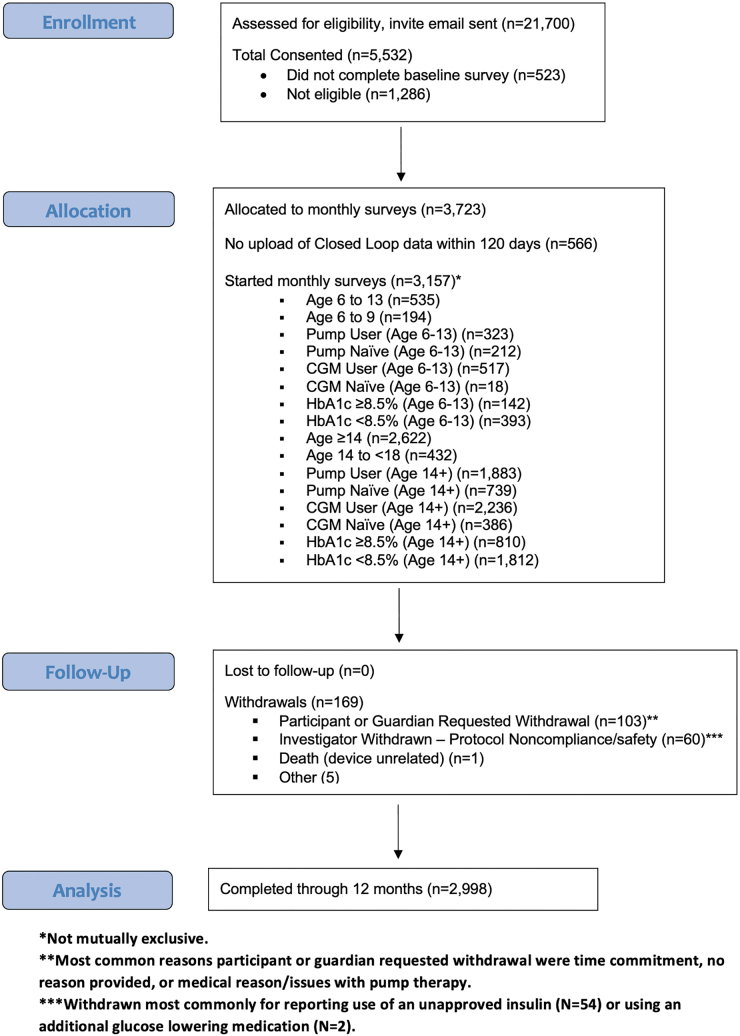
Participant allocation during the study.

Five thousand five hundred thirty-two individuals signed the consent form. Three thousand seven hundred twenty-three individuals were deemed eligible on the baseline survey and enrolled in the study. Participants were excluded based on results of the baseline survey most commonly because the protocol was already fully enrolled for age/prior A1c/device use category, for not having a recent A1c value, or not using an approved insulin with the pump. Five hundred sixty-six individuals never uploaded closed-loop data within 120 days and per protocol were considered screen failures. Therefore, a total of 3157 individuals began the monthly surveys. This included 191 individuals with their last HbA1c drawn more than 6 months before enrollment who were allowed to enroll due to being unable to obtain a recent A1c during a peak of the COVID emergency. Two thousand nine hundred ninety-eight participants completed the study (Fig. 2).

Of the 3157 participants who began the monthly surveys and were followed for 12 months, median participant age was 29.0 (16.0–45.0) years. 55.7% were female. Last reported A1c before enrollment was 7.7 (6.9–8.7)% [61 (52–72) mmol/mol]. Two thousand one hundred ninety participants (69.4%) were adults. 38.4% were prior Tandem pump users, 31.5% prior pump users of a different brand, and 30.1% were prior MDI users. The vast majority (87.2%) reported at least some prior CGM experience. Full participant demographics are listed in Table 1. Baseline A1c and race/ethnicity demographics were very similar to participants in prior Control-IQ RCTs.^3^

**Table 1.** Participant Demographics for Those Who Started the Monthly Surveys (*N* = 3157)

Age (years), median (IQR)	29.0 (16.0–45.0)
HbA1c (%), median (IQR)	7.7 (6.9–8.7)
BMI (kg/m^2^), median (IQR)	25.7 (21.9–30.2)
Total daily insulin (units/day), median (IQR)	49.9 (35.6–69.0)
Gender: Female, *n* (%)	1759 (55.7)
Race/ethnicity, *n* (%) (not mutually exclusive)	
White non-Hispanic	2628 (83.2)
Hispanic or Latino	271 (8.6)
Black/African American	184 (5.8)
Asian	78(2.5)
American Indian/Alaskan Native	60 (1.9)
Native Hawaiian or Pacific Islander	14 (0.4)
Unknown/not reported	67 (2.1)
Participant income, *n* (%)	
<$10,000	629 (19.9)
$10,000 to <$25,000	292 (9.2)
$25,000 to <$75,0000	868 (27.5)
$75,000 to <$100,00	288 (9.1)
$100,000 to <$150,000	268 (8.5)
$150,000 or greater	176 (5.6)
Unknown/not reported	636 (20.1)
Participant highest education level, *n* (%)	
Less than high school degree	877 (27.8)
High school graduate	291 (9.2)
Some college but no degree	540 (17.1)
Associate degree in college (2-year)	286 (9.1)
Bachelor's degree in college (4-year)	735 (23.3)
Master's degree	326 (10.3)
Doctoral degree	49 (1.6)
Professional degree (JD, MD)	53 (1.7)
Unknown/not reported	0 (0.0)
Insulin modality, *n* (%)	
MDI	951 (30.1)
Tandem pump	1212 (38.4)
Other brand pump	994 (31.4)
CGM user, *n* (%)	2753 (87.2)

BMI, body mass index; CGM, continuous glucose monitoring; HbA1, hemoglobin A1c; IQR, interquartile range; MDI, multiple daily injections.

For this study, 85.1% of prior Tandem pump users performed a software update to obtain Control-IQ technology. These users self-trained using the Tandem online portal for their pump training. In addition, 2248 participants (71.2%) used the t:connect mobile app at some point during the study, allowing them to upload all data from their pump without the need for a personal computer and USB cable.

### Adverse events

AE rates (SH, DKA) with use of Control-IQ technology over the 12 months period were significantly lower for both adults and children compared to historically reported data (Table 2). One thousand one hundred ninety-four surveys with AE's were ultimately determined from call center data and follow-up contacts to not require assistance for treatment or no change in mental status for SH, not requiring hospitalization and/or not diagnosed with DKA, and/or no evidence of the event on t-connect or other supporting data. These events were excluded. No events were excluded or reclassified by the quarterly independent review.

**Table 2.** Adverse Event Rates

AE rates	CIQ rate	Historical rate	Effect size,* P*-value
Age 18+ (*N* = 2130)			
SH	9.77 (36.97)	29.49 (104.54)	*d* = 0.53, *P* < 0.01
DKA	1.46 (13.10)	9.81 (61.87)	*d* = 0.64, *P* < 0.01
Age 6–17 (*N* = 931)			
SH	9.31 (34.31)	19.31 (85.73)	*d* = 0.29, *P* < 0.01
DKA	1.93 (13.78)	12.81 (70.43)	*d* = 0.79, *P* < 0.01

Mean (SD) AE rates per 100 patient years for study participants as self-reported on monthly surveys through 12 months of Control-IQ technology use, compared to historical rates. Both adults and children showed a lower observed rate of self-reported AEs with Control-IQ technology use compared to historical data from the T1D exchange. Effect size for *t*-test between groups was determined by Cohen's d. Significance was defined as *P*-value <0.05. Age groups for comparison were determined by how historical data were reported by Foster et al.^2^

AE, adverse event; CIQ, Control-IQ Technology; DKA, diabetic ketoacidosis; SH, severe hypoglycemia.

For adults, the observed rate of SH was 9.77 (36.97) for Control-IQ use versus 29.45 (104.54) for the historical data from the T1D Exchange (effect size *d* = 0.53, *P* < 0.01), while for children, the observed rate of SH was 9.31 (34.31) for Control-IQ use versus 19.31 (85.73) for the historical data (effect size *d* = 0.29, *P* < 0.01). For adults, the observed rate of DKA was 1.46 (13.10) for Control-IQ use versus 9.81 (61.87) for the historical data from the T1D Exchange (effect size *d* = 0.64, *P* < 0.01), while for children, the observed rate of DKA was 1.93 (13.78) for Control-IQ use versus 12.81 (70.43) for the historical data (effect size *d* = 0.79, *P* < 0.01).

The lower observed rate of AEs compared to historical data was independent of baseline HbA1c, prior insulin delivery method (pump vs. MDI), or prior CGM experience, with all groups showing lower AE rates (Supplementary Table S1). AE rates broken down further by age 6–13, 14–17, and age 18+ are listed in Supplementary Table S2.

### Glycemic outcomes

Median time in range 70–180 mg/dL (3.9–10.0 mmol/L) was 70.1% (61.0–78.8) for adults, and above 60% for both children and adolescents, meeting international consensus guidelines for time in range across all age groups^13,14^ (Table 3). Time in hypoglycemia was very low across the study.

**Table 3.** Glycemic Outcomes

Glycemic outcomes	Median (IQR)
Age ≥18 (*N* = 2130)	
% Time 70–180 mg/dL	70.1 (61.0–78.8)
% Time >180 mg/dL	28.6 (19.2–37.8)
% Time >250 mg/dL	6.3 (2.8–12.0)
% Time <70 mg/dL	1.1 (0.5–2.1)
% Time <54 mg/dL	0.2 (0.1–0.4)
Mean glucose	158.0 (144.5–172.6)
Age 14–17 (*N* = 412)	
% Time 70–180 mg/dL	60.9 (50.1–71.8)
% Time >180 mg/dL	37.0 (26.5–48.7)
% Time >250 mg/dL	12.8 (6.1–21.9)
% Time <70 mg/dL	1.0 (0.5–1.9)
% Time <54 mg/dL	0.2 (0.1–0.4)
Mean glucose	171.4 (153.8–192.3)
Age 6–13 (*N* = 519)	
% Time 70–180 mg/dL	61.2 (52.4–70.5)
% Time >180 mg/dL	37.2 (27.5–46.3)
% Time >250 mg/dL	12.7 (7.5–21.0)
% Time <70 mg/dL	1.1 (0.6–2.2)
% Time <54 mg/dL	0.2 (0.1–0.4)
Mean glucose	171.9 (156.5–189.6)
Overall (*N* = 3061)	
% Time 70–180 mg/dL	67.3 (57.4–76.9)
% Time >180 mg/dL	31.4 (20.9–41.5)
% Time >250 mg/dL	8.0 (3.6–14.8)
% Time <70 mg/dL	1.1 (0.5–2.1)
% Time <54 mg/dL	0.2 (0.1–0.4)
Mean glucose	161.7 (147.3–178.6)

Glycemic outcomes for each age cohort and overall with 12 months of Control-IQ technology use.

Evaluation of autopopulation of CGM values into the bolus calculator is shown in Supplementary Table S3. The percentage of boluses using the autopopulation feature resulted in fewer readings <54 mg/dL (<3.0 mmol/L) and <70 mg/dL (<3.9 mmol/L) than those not using the feature, in every prebolus glucose range examined. This was examined for glucose ranges before bolus of 70–180 mg/dL, 181–250 mg/dL, >250 mg/dL, and overall. There was no evidence of increased risk of hypoglycemia when autopopulated CGM results were used to calculate the subsequent bolus versus manual entry of glucose levels into the bolus calculator.

### PRO results

Two thousand seven hundred seventy-eight participants completed the month 12 survey (Supplementary Table S4). In the overall cohort, the DIDS Device Satisfaction median score increased from 7.29 (5.57–8.71) at baseline to 9.14 (8.29–9.71) at 12 months, while the Diabetes Impact median score was 4.75 (3.25–6.0) at baseline and 2.75 (2.0–4.0) at 12 months (*P* < 0.01). This represents a 25% increase in device satisfaction and a 42% reduction in diabetes impact. Notably, device satisfaction increased in both participants transitioning to Control-IQ from a different pump system [6.29 (5.0–7.43) to 9.0 (8.14–9.71)] and for participants transitioning from MDI therapy [5.86 (4.71–7.0) to 9.14 (8.29–9.71)] (*P* < 0.01). Similarly, Diabetes Impact scores decreased for both participants transitioning from another pump system [4.75 (3.5–6.25) to 2.75 (2.0–3.75)] and for participants transitioning from MDI therapy [5.25 (4.0–6.5) to 3.0 (2.0–4.0)] (*P* < 0.01).

The DIDP scores indicated a reduced impact of diabetes on quality of life, with scores improving from 4.86 (4.43–5.29) at baseline to 4.67 (4.14–5.0) (*P* < 0.01) at 12 months. Self-reported quality of sleep, which was a 5-point scale, was a median of 3.0 (2.0–3.0) at baseline and 4.0 (3.0–4.0) for the overall cohort at 12 months (*P* < 0.01).

A post hoc analysis of the 191 participants whose last HbA1c level was drawn greater than 6 months before enrollment showed that they were much more likely to have had concerns about COVID-19 (*P* < 0.001), and were much more likely to have obtained Control-IQ technology through a remote software update than the rest of the cohort (*P* = 0.001).

## Conclusions

Recent data from the T1D exchange (2016–2018) show that most individuals with T1D in the United States are not meeting glycemic goals.^2^ At the same time, long-term follow-up data from the Diabetes Control and Complications Trial (DCCT) have shown that one half of the DCCT/Epidemiology of Diabetes Interventions and Complications cohort reported episodes of SH, with SH rates of 35–40 events per 100 patient years,^15^ further emphasizing the risks of intensive insulin treatment.

Our analysis shows lower observed AE rates for both SH and DKA with use of t:slim X2 with Control-IQ technology over 12 months compared to historic rates. Concurrent with these lower rates, on average adult participants achieved >70% CGM time in range, while pediatric participants achieved >60% CGM time in range, achieving international consensus guidelines for time-in-range.^13,14^ The study also showed safety of autopopulation of CGM results into the bolus calculator, with no increased risk of hypoglycemia after boluses given with autopopulated CGM results versus manual entry of glucose values.

Prior studies of Control-IQ technology have shown significant improvements in sensor time in range metrics. Randomized, controlled clinical trials showed a mean adjusted 11% improvement in time in range for both adolescents and adults (age 14+) and children (age 6–13 years), and 12% mean adjusted improvement compared to the control group for preschoolers, compared to sensor-augment pump or standard care.^4–6^ Early real-world results in 1435 individuals age 14+ onboarding to Control-IQ technology showed achievement of time in range to 79.2% overall after 7 weeks of use, with very low rates of time below range for these early adopters.^10^ Subsequently, data from outside of the United States has been published, supporting significant time in range improvements for children and adolescents adopting Control-IQ technology.^16,17^ AE rates were also reported to be very low across these trials.

During the Free-Life Kid AP trial of 122 prepubertal children, neither DKA nor SH events occurred while Control-IQ technology was active for a period of 36 weeks in free living conditions.^17^ During that trial, participants achieved substantially better outcomes with Control-IQ technology than sensor augmented pump on days with missed meal boluses, demonstrating the benefit of AHCL in this population.^18^

The unique contribution of the CLIO study is the virtual nature of all follow-ups, which, even though designed as such, was forced, in part, by performing the study during the peak of the COVID-19 emergency. Prior AHCL postmarket studies were performed in the traditional manner, with mandated in clinic visits, regular supervision and recruitment at expert clinical sites (such as NCT02748018). However, a recent pivotal study of Control-IQ technology was performed with over 90% virtual visits,^6^ suggesting larger trials with primarily remote visits are possible.

The recent Loop real-world prospective study of 558 adults and children who initiated Loop either on their own or with community-developed resources and were followed for 6 months was also performed with all virtual visits, attesting to the feasibility of performing large device trials in a decentralized manner.^19^ In that 6-month study, 35 (6%) of participants experienced a total of 51 confirmed SH events (incidence rate 18.7 per 100 person years), an observed rate of SH lower than the T1D exchange data. There were no DKA events.

In our study, participants onboarded to Control-IQ technology through their regular diabetes care provider, allowing participants to be trained at the point of convenience, which may have been a concurrent medical appointment or virtually, as previously described.^20^ With a large mix of participants with differing age, baseline HbA1c, prior therapy modality, as well as varied method of training (such as self-training after a software update), this data set is differentiated from those collected during prior large postmarket device clinical trials and other recent and ongoing postmarketing device studies in that it reflects representative real-world training and use in a broad variety of settings.^21^ This study showed that people who began use of Control-IQ technology and continued for 1 year experienced similar outcomes as those who participated in prior clinical trials, despite ongoing stresses related to the COVID-19 pandemic that has been reported in this population.^10^

While the CLIO study did not directly measure costs associated with AEs, Bullano et al. estimated the proportion of hypoglycemia resulting in either an ER visit alone or an ER visit followed by an inpatient hospitalization to be 17% versus 24%, respectively.^22^ Using a large national US payer claims data set, Liu et al. estimated that 1/3 of individuals with T1D admitted for a SH event were subsequently readmitted for SH, with an estimated cost of inpatient hospitalization to be $3915 in 2021 dollars.^23^ AHCL use presents an opportunity to alleviate patient burden associated with AE's as well as improve access and potentially limit corresponding medical resource utilization and cost.

PROs in this study indicate a positive effect of t:slim X2 with Control-IQ technology on the burden of diabetes care for participants. These brief measures adequately highlight the reduction in burden, including increase in insulin delivery device satisfaction, decrease in diabetes impact on quality of life, and increase in sleep quality. Further, the participants were more satisfied using this AHCL system than their baseline insulin delivery method, for both prior pump users of a different brand and for prior MDI users. Both the single item sleep question and the DIDS Diabetes Impact factor assessed subjective sleep quality. Sleep disturbances are common for people living with diabetes, and sleep quality and quantity are associated with glycemic outcomes.^24^

The impact of technology on sleep has been mixed,^25^ with some studies indicating AHCL to be neutral or detrimental to sleep quality,^24,26,27^ while others indicate a positive impact on sleep quality.^10,28,29^ The sleep items assessed in this study all indicate improvement in sleep quality with Control-IQ technology, which also coincides with the improved TIR metrics overnight achieved with the Control-IQ system. The synergistic effect of improved overnight glycemia and sleep quality have been tied to improved daytime behavior and executive functioning in children with diabetes,^30^ and improved daily functioning in adults as well.

Strengths of the study include a large sample size, and the fact that the study was performed in the real-world outpatient setting, without extra supervision from clinical sites as is typically performed in postmarket device studies to better reflect real-world use. The study included a much larger cohort of prior MDI users than in previous trials, and these participants showed the greatest improvement in lowering the observed rate of SH (Supplementary Table S1). The virtual nature of all follow-up visits also proved that diabetes device studies, which can involve complicated devices such as AHCL systems, can move toward a model of decentralized clinical trials, as discussed in FDA's recent guidance.^31^ This study enrolled a diverse group of participants, which may not have been possible if the study recruited only in person.

In fact, given that participants who did not have a recent HbA1c completed within the last 6 months also reported higher levels of concern related to COVID, and were more likely to obtain Control-IQ through a software update and perform training only on the Tandem portal website, we can speculate that these participants would not have been likely to join a traditional clinical trial with in-person visits. The study also allowed use of tools that facilitated automated data collection, such as the t:connect mobile app, which 71.2% of participants eventually used. This enabled completion of this postmarket study in under 3 years, faster than average.^32^

This study had limitations. First, it used different methodology than prior studies to define SH and DKA, potentially increasing reported AE rates in our study. Rates for these AE's as determined by T1D exchange surveys required loss of consciousness or seizure for SH, or overnight hospitalization for DKA,^2^ more stringent than our requirements. AE's were also self-reported, which could lead to inaccuracies in the reported information. Baseline survey AE's were not verified, and thus could not be used for comparison by the same methodology.

The study did not include a run-in period of blinded CGM before Control-IQ use, or a control group, that would have offered a better comparison for AE rates. Individuals today are able to choose a variety of automated insulin delivery systems and CGMs that were not available during the time period of the T1D Exchange historical cohort. As such, there is a limitation in directly comparing rates to a cohort with a differing set of baseline therapy choices. In addition, there was a wide range for the standard deviation of reported events in all categories for both the historical data and Control-IQ use. Finally, the question of a “digital divide” of who could participate and upload remotely, and whether this could exclude individuals, remains open.

We conclude that use of the Tandem t:slim X2 insulin pump with Control-IQ technology is safe and effective in individuals with T1D. Recognizing the rapid pace of innovation and technological change in AHCL devices, future postmarket studies may need to follow the model of CLIO with virtual visits to ensure timely completion and improve access to allow for broad participation.

## Supplementary Material

Supplemental data

Supplemental data

Supplemental data

Supplemental data

Supplemental data
