# Supplementary material for: Real-World Use of Control-IQ Technology Is Associated with a Lower Rate of Severe Hypoglycemia and Diabetic Ketoacidosis Than Historical Data: Results of the Control-IQ Observational (CLIO) Prospective Study
Source: Diabetes Technol Ther. 2024 Jan 5;26(1):24–32. doi: 10.1089/dia.2023.0341 (PMC10794820; doi:10.1089/dia.2023.0341)
Supplement: Supplemental data [file Suppl_Data.zip › SupplementalMaterialAdditionalPowerAnalysisDetails.pdf]

Additional Details of Power Analysis

A separate power analysis for the 14-<18 age group used an overall SH incidence rate of 19.5 events/100 patient years (PY). Results indicated that a sample size of 231 individuals with T1D between the ages of 14-18 would provide 80% power with a type 1 error rate (two-sided) of 5% to detect a difference, if there is one, between the expected SH rate (19.5) and the proposed study sample SH event rate. Of the overall sample of 1,354 subjects, at least 231 subjects will be 14-18 years old to provide sufficient power to detect risk of SH in this younger age group.

A separate power analysis for the 6-13 year old group used a SH incidence rate of 21.9 events/100 PY, which is a weighted average of both MDI and pump users (since pump users were overrepresented in this age group). Results indicated that a sample size of 288 individuals with T1D between the ages of 6 and 13 would provide 80% power with a type 1 error rate (two-sided) of 5% to detect a difference, if there is one, between the expected SH rate (21.9) and the proposed study sample SH event rate. In addition to the overall sample, at least 288 subjects will be 6-13 years old to provide sufficient power to detect risk of SH in this younger age group.

A separate power analysis for the 14-<18 age group used an overall DKA incidence rate of 14.2 events/100PY. Results indicated that a sample size of 328 individuals with T1D between the ages of 14-<18 would provide 80% power with a type 1 error rate (two-sided) of 5% to detect a difference, if there is one, between the expected DKA rate (14.2) and the proposed study sample DKA event rate. Of the overall sample of 1,282 subjects, at least 328 subjects will be 14-<18 years old to provide sufficient power to detect risk of DKA in this younger age group.

A separate power analysis for the 6-13 year old group used an overall DKA incidence rate of 13.2 events/100 PY, which is a weighted average of both MDI and pump users (since

pump users were overrepresented in this age group). Results indicated that a sample size of 77 individuals with T1D between the ages of 6-13 would provide 80% power with a type 1 error rate (two-sided) of 5% to detect a difference, if there is one, between the expected DKA rate (13.2/100PY) and the proposed study sample DKA event rate. In addition to the previously determined sample, at least 77 subjects will be 6-13 years old to provide sufficient power to detect risk of DKA in this younger age group.
