# Supplementary material for: Real-World Use of Control-IQ Technology Is Associated with a Lower Rate of Severe Hypoglycemia and Diabetic Ketoacidosis Than Historical Data: Results of the Control-IQ Observational (CLIO) Prospective Study
Source: Diabetes Technol Ther. 2024 Jan 5;26(1):24–32. doi: 10.1089/dia.2023.0341 (PMC10794820; doi:10.1089/dia.2023.0341)
Supplement: Supplemental data [file Suppl_Data.zip › SupplementalMaterialSurveyBaseline.pdf]

TANDEM®
DIABETES CARE

Inline Email Question

Would you like to learn more about this research study and participate in the first survey?

- ◯ Yes
- ◯ No

Confirm Negative

You indicated that you are not interested in learning more about the opportunity to participate in the online Tandem research study. Please confirm by selecting one option below:

- ◯ I would like to learn more about the research study.
- ◯ I am NOT interested in learning more about the research study, please exclude me from any other emails related to this research study.

CA Bill of Rights

California Experimental Subject's Bill Of Rights

Any person who is requested to consent to participate as a subject in a research study involving a medical experiment, or who is requested to consent on behalf of another has the right to:

(a) Be informed of the nature and purpose of the experiment.

(b) Be given an explanation of the procedures to be followed in the medical experiment and any drug or device to be used.

(c) Be given a description of any attendant discomforts and risks reasonably to be expected from the experiment, if applicable.

(d) Be given an explanation of any benefits to the subject reasonably to be expected from the experiment if applicable.

(e) Be given a disclosure of any appropriate alternative procedures, drugs, or devices that might be advantageous to the subject, and their relative risks and benefits.

(f) Be informed of the avenues of medical treatment, if any, available to the subject after the experiment or if complications should arise.

(g) Be given an opportunity to ask any questions concerning the experiment or other procedures involved.

(h) Be instructed that consent to participate in the medical experiment may be withdrawn at any time, and the subject may discontinue in the medical experiment without prejudice.

(i) Be given a copy of a signed and dated written informed consent form when one is required.

(j) Be given the opportunity to decide to consent or not to consent to a medical experiment without the intervention of any element of force, fraud, deceit, duress, coercion or undue influence on the subject's decision.

Signature of adult subject capable of consent, child subject’s parent, individual authorized to consent to the child subject’s general medical care, or adult subject’s legally authorized representative:

SIGN HERE

×

clear

Date:

Print a copy of this form for your records by selecting the button below:

Print this page

Screener_Age

First, we have a few questions to check your eligibility to participate in this study.

What is your age? (in years)

[▾]

Screener

What type of diabetes do you have?

- ○ Type 1 diabetes
- ○ Type 2 diabetes
- ○ Gestational diabetes
- ○ None of the above

Please read the list of statements below and indicate whether the statement applies to you by selecting 'yes' or 'no'.

	Yes	No
I am a health care provider.	○	○
I am a current employee (full-time or part-time) of Tandem Diabetes Care.	○	○
I am currently pregnant or plan to become pregnant in the next 12 months.	○	○

Thank you for your interest but current full-time or part-time Tandem employees are not eligible to participate in this study.

Healthcare professionals, including physicians, nurses, and other individuals who have the ability to prescribe are not eligible for this study. We are unable to provide study compensation to any healthcare provider that is or may be subject to federal or state Sunshine Act/Open Payment laws.

In order to participate in this study, we will require that your pump data is uploaded to the t:connect web application.

For the duration of this study, you can choose to either:

a. Download the t:connect mobile app on your phone and enable automatic upload of your pump data to the t:connect web application. **CLICK TO LEARN HOW TO DO THIS>**

- OR -

b. Connect your Tandem pump to your computer via USB in order to upload your data to the t:connect web application. **CLICK TO LEARN HOW TO DO THIS>**

Please indicate how you prefer to meet this study requirement by selecting one of the options below:

- ○ I will download the t:connect mobile app
- ○ I will upload my data via USB using the t:connect web application
- ○ I am not sure yet, but I will do either option
- ○ I am not willing to download the t:connect mobile app or upload my data to the t:connect web application

Based on your response to the question above, you are not eligible to participate in this study because you do not meet the following study requirement:

"Willingness to download the t:connect mobile application to your smartphone and keep it active throughout the study. If unable to use the t:connect mobile application, must be willing to upload your pump data to the t:connect web application by connecting your Tandem pump to your computer via USB a least once every three months and at the completion of the study"

If you would like to change your response, please do so now.

If you are not willing to have your pump data in the t:connect web application for the duration of the study, click the opt out button below to withdraw from this research study.

○ **Opt out and exit survey**

Previous Method of Diabetes Management Before Control-IQ

These next few questions ask about how you have been managing your diabetes over the **last 3 months.**

Have you used a continuous glucose monitor (CGM) in the last three months?

○ Yes
○ No

What type of CGM?

○ Dexcom G6 CGM
○ Dexcom G5 Mobile CGM
○ Dexcom G4 PLATINUM CGM
○ Medtronic CGM

- ○ Abbott Freestyle Libre
- ○ Senseonics Eversense Implantable CGM
- ○ [ ] Other

How long have you used a CGM?

- ○ Less than 1 month
- ○ 1-6 months
- ○ 7-12 months
- ○ 1-2 years
- ○ 2-4 years
- ○ 5-9 years
- ○ 10+ years

In the **last 3 months,** what has been your **primary** method of insulin delivery?

- ○ Multiple Daily Injections (e.g., insulin pen, syringe)
- ○ Animas Insulin Pump
- ○ Insulet OmniPod Insulin Pump
- ○ Medtronic MiniMed Insulin Pump
- ○ Tandem Insulin Pump

○ Roche Accu-Chek Insulin Pump

○ DIY or Open APS System

○ [ ] Other

You selected Multiple Daily Injections as your primary method of insulin delivery. In the last 3 months, have you used syringes or pens?

○ Syringes

○ Pens

○ Both syringes and pens

Which Medtronic MiniMed insulin pump have you been using?

MiniMed 670G System

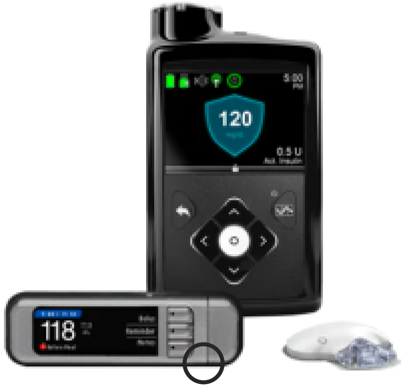

○

MiniMed 630G System

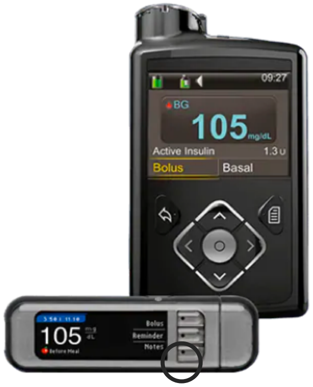

○

MiniMed 530G, Revel Paradigm (or older model)

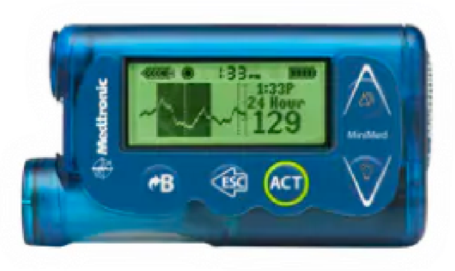

○

How long have you used your?

- ○ Less than 1 month
- ○ 1-6 months
- ○ 7-12 months
- ○ 1-2 years
- ○ 2-3 years
- ○ 3-4 years
- ○ 4+ years

When did you last have your HbA1c test done? If you are unsure, please take your best guess.

- ○ In the past 2 weeks
- ○ In the last month
- ○ In the last 2 - 3 months
- ○ In the last 4 - 6 months
- ○ In the last 6 - 12 months
- ○ Longer than 1 year ago

What was your most recent HbA1c value? If you are unsure, please take your best guess.

Sleep Question

The following question is about your quality of sleep.

Thinking about the past month, how would you rate your sleep quality?

Very Poor	Poor	Average	Good	Very Good
○	○	○	○	○

Diabetes Impact and Devices Satisfaction Scale

The following questions ask about your experience in the last 3 months using your.

How satisfied are you with your

Very **Unsatisfied**									Very **Satisfied**
1	2	3	4	5	6	7	8	9	10
○	○	○	○	○	○	○	○	○	○

How much do you trust your?

**Not at all**									**A lot**
1	2	3	4	5	6	7	8	9	10
○	○	○	○	○	○	○	○	○	○

Please indicate how much you agree or disagree with each statement based on your experience using your.

My..

	Strongly **Disagree** 1	2	3	4	5	6	7	8	9	Strongly **Agree** 10
...is easy to use.	○	○	○	○	○	○	○	○	○	○
...helps me have good blood glucose control.	○	○	○	○	○	○	○	○	○	○

	Strongly **Disagree** 1	2	3	4	5	6	7	8	9	Strongly **Agree** 10
...is a hassle to use.	○	○	○	○	○	○	○	○	○	○
...helps me feel more in control of my diabetes.	○	○	○	○	○	○	○	○	○	○
...is too complicated.	○	○	○	○	○	○	○	○	○	○

In the last 3 months, how often did you....

	**Never** 1	2	3	4	5	6	7	8	9	**Always** 10
Have a bad night's sleep because of diabetes?	○	○	○	○	○	○	○	○	○	○
Wake up at night to treat low blood glucose?	○	○	○	○	○	○	○	○	○	○
Miss work, school, household chores, or other responsibilities due to diabetes?	○	○	○	○	○	○	○	○	○	○
Worry about going low?	○	○	○	○	○	○	○	○	○	○

Impact of Diabetes profile

How does diabetes CURRENTLY impact the following aspects of your life?
Select one option on each line. Only use the N/A option if the statement is not applicable to you.

	Very negative impact	Negative impact	Slightly negative impact	No impact	Slightly positive impact	Positive impact	Very positive impact	N/A
Your physical health	○	○	○	○	○	○	○	○
Your financial situation	○	○	○	○	○	○	○	○
Your relationship with your family, friends and peers	○	○	○	○	○	○	○	○
Your leisure activities	○	○	○	○	○	○	○	○
Your work or studies	○	○	○	○	○	○	○	○
Your emotional well-being	○	○	○	○	○	○	○	○
Your freedom to eat as you wish	○	○	○	○	○	○	○	○

AE Questions

This section will include questions about any diabetes complications you have had in the ***last 3 months*** *while using your.*

First, we would like to ask you about severe hypoglycemia (low blood sugar) episodes.

> Definition of Severe Hypoglycemia:
> A severe hypoglycemia episode is a situation in which your blood sugar is low, and you need help from another person to help you raise your blood sugar by giving you sugar, carbs, glucagon or calling 911. The other person might be a friend or family member, or a healthcare professional.

In the last 3 months, did you have any severe hypoglycemia episodes that were treated with help from another person (e.g., friend, family member, medical/healthcare provider)?

- ○ [ ] Yes
- ○ No

You indicated that you had severe hypoglycemia episodes in the last 3 months. How many required a trip to the

emergency room or hospital?

These next few questions will ask about any episodes of diabetic ketoacidosis (DKA) you may have had in the last 3 months.

Definition of Diabetic Ketoacidosis (DKA): DKA is a situation in which your blood sugar is high and each of the following occurred:

1. You felt sick to your stomach (with or without vomiting), and/or you were urinating more than usual and/or more thirsty than usual
2. You were treated in a health care facility
3. You were told by a health care provider that you had diabetic ketoacidosis or DKA.

In the last 3 months, did you have any Diabetic Ketoacidosis (DKA) episodes, as defined above?

- ○ Yes
- ○ No

You indicated that you had DKA episodes in the last 3 months. How many required a trip to the emergency room or hospital?

Baseline Demographics

This section includes general demographic questions.

Which of the options best applies to you in terms of diabetes? You may select more than one.

- ☐ Person with diabetes
- ☐ A parent or guardian of someone with diabetes
- ☐ A spouse or partner of someone with diabetes
- ☐ A caregiver of someone with diabetes
- ☐ Other

In the last 3 months, what type(s) of insulin have you used with? (Please select all that apply.)

- ☐ Humalog (U-100)
- ☐ Humalog (U-200)
- ☐ Novolog
- ☐ Apidra
- ☐ Lantus
- ☐ Fiasp
- ☐ Levemir
- ☐ Tresiba
- ☐ Other (please specify): ____________

In the last 3 months, what is the average number of insulin units you used per day? If you are unsure, please take your best guess.

Average # of Insulin Units Per Day: ____________

What is your current height?

What is your current weight? (pounds)

Are you of Hispanic/Latino/Spanish origin?

- ○ Yes
- ○ No
- ○ Prefer not to answer

Choose one or more races that you consider yourself to be:

- ☐ American Indian or Alaska Native
- ☐ Black or African American
- ☐ Asian
- ☐ White
- ☐ Native Hawaiian or Pacific Islander
- ☐ Prefer not to answer

- [ ] ______ Other

What is your current relationship status?

- ○ Widowed
- ○ Living with a partner
- ○ Prefer not to answer
- ○ Married
- ○ Divorced
- ○ Never Married
- ○ Separated

What is the highest level of school you have completed or the highest degree you have received?

- ○ Less than high school degree
- ○ High school graduate (high school diploma or equivalent including GED)
- ○ Some college but no degree
- ○ Associate degree in college (2-year)
- ○ Bachelor's degree in college (4-year)
- ○ Master's degree

- ○ Doctoral degree
- ○ Professional degree (JD, MD)

Which of these describes your annual income?

- ○ Less than $10,000
- ○ $10,000 - $24,999
- ○ $25,000 - $74,999
- ○ $75,000 - $99,999
- ○ $100,000 - $149,999
- ○ $150,000 and greater
- ○ Prefer not to answer

Some people may experience other health complications while they are managing their diabetes. Please indicate if you are currently undergoing treatment or taking medications for any of the following.

- ☐ Eye problems
- ☐ Kidney problems
- ☐ Dental problems
- ☐ Numbness/tingling/loss of sensation in your feet
- ☐ High blood pressure

- ☐ High cholesterol
- ☐ Sexual problems
- ☐ Depression
- ☐ Anxiety
- ☐ None
- ☐ Prefer not to answer

On average, how often do you exercise in a week?

- ○ Once a week
- ○ 2-3 times per week
- ○ 4-5 times per week
- ○ 6+ times per week
- ○ I do not exercise regularly

Do you currently use a smartphone?

- ○ ☐ Yes, I use an iPhone.
- ○ ☐ Yes, I use an Android.
- ○ No, I do not use a smartphone.

COVID-19 Questions

This last section includes questions about the Coronavirus (COVID-19) disease.

With regards to the current Coronavirus (COVID-19) situation, how concerned are you about each of the following:

	Not At All Concerned	Not Concerned	Neutral	Concerned	Very Concerned
The possible impact of the virus on your family and/or friends	○	○	○	○	○
How it might affect your diabetes	○	○	○	○	○
The possible impact of the virus on your overall health	○	○	○	○	○
Your ability to get diabetes supplies	○	○	○	○	○

Have you ever been diagnosed with Coronavirus (COVID-19)?

○ Yes

- [ ] No
- [ ] Prefer not to answer

Are you/were you caring for someone diagnosed with Coronavirus (COVID-19)?

- [ ] Yes
- [ ] No
- [ ] Prefer not to answer

Open End Question

Is there anything else you would like to tell us about your experience with using? Please explain in the text box below.
