# Supplementary material for: Real-World Use of Control-IQ Technology Is Associated with a Lower Rate of Severe Hypoglycemia and Diabetic Ketoacidosis Than Historical Data: Results of the Control-IQ Observational (CLIO) Prospective Study
Source: Diabetes Technol Ther. 2024 Jan 5;26(1):24–32. doi: 10.1089/dia.2023.0341 (PMC10794820; doi:10.1089/dia.2023.0341)
Supplement: Supplemental data [file Suppl_Data.zip › SupplementalMaterialSurveyMonth12andPROs.pdf]

TANDEM® DIABETES CARE

CLIO Study: Month 12 Feedback Survey

## Introduction

You are about to start the Month 12 Feedback Survey, which contains questions about your experience using Control-IQ™ technology and any diabetes complications you may have experienced.

Once you complete this survey, you will receive a $50 Reward Card. With your Reward Card, you can choose from an assortment of e-gift cards, prepaid cards, digital checks, and donations.

Select the **NEXT** button to get started.

## CIQ Use

How frequently do you have the Control-IQ technology feature turn on? (meaning, the toggle button is selected on)

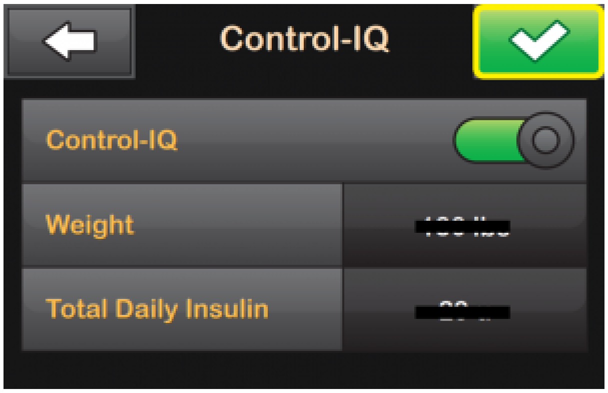

- ○ Always
- ○ Most of the time
- ○ About half the time
- ○ Sometimes
- ○ Never

When is it most important for you to have your Control-IQ technology turned on? Select all that apply.

- ☐ During the day
- ☐ At night, when sleeping
- ☐ During exercise
- ☐ [ ] Other

You indicated that you never have the Control-IQ feature turned on.

**Are you still using your t:slim X2 insulin pump?**

- ○ Yes, I am still using my t:slim X2 insulin pump.
- ○ No, I stopped using my t:slim X2 insulin pump.

Please indicate why you never turn on the Control-IQ feature on your pump. Select all that apply.

- ☐ I ran out of CGM supplies and/or unable to get a CGM sensor.
- ☐ I chose not to wear a CGM sensor.
- ☐ Control-IQ was not helping me reach my desired blood glucose targets.
- ☐ It had too many glitches and bugs.
- ☐ Control-IQ technology took too long to respond to high blood glucose

- [ ] Control-IQ technology took too long to respond to low blood glucose
- [ ] ______ Other

From the list below, please select the reason(s) why you stopped using your t:slim X2 pump. Select all that apply.

- [ ] Problems with insertion/adhesive
- [ ] It was too expensive
- [ ] It interfered with sports activities
- [ ] It was uncomfortable to wear
- [ ] Disliked wearing the pump
- [ ] It interfered with intimacy
- [ ] The pump was not working properly
- [ ] The pump was not helping me reach my desired blood glucose targets
- [ ] The pump was difficult to use
- [ ] The pump is not waterproof
- [ ] Did not trust it
- [ ] I wanted a break from wearing my pump
- [ ] I was unable to get the pump and/or CGM supplies I needed
- [ ] ______ Other

Since you have stopped using your Tandem pump, what has been your primary method of insulin delivery?

- ○ Multiple Daily Injections (e.g., insulin pen, syringe)
- ○ Animas Insulin Pump
- ○ Insulet OmniPod Insulin Pump
- ○ Medtronic MiniMed Insulin Pump

○ Roche Accu-Chek Insulin Pump
○ DIY or Open APS System
○ [ ] Other

You selected Multiple Daily Injections as your primary method of insulin delivery. In the last 3 months, have you used syringes or pens?

○ Syringes
○ Pens
○ Both syringes and pens

Which Medtronic MiniMed insulin pump have you been using?

MiniMed 670G System

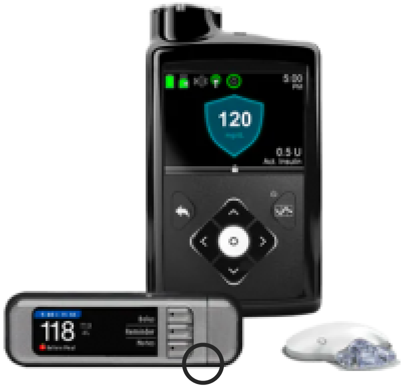


○

MiniMed 630G System

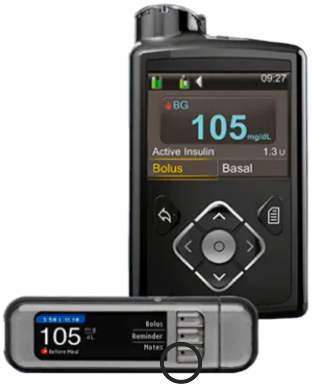


○

MiniMed 530G, Revel Paradigm (or older model)

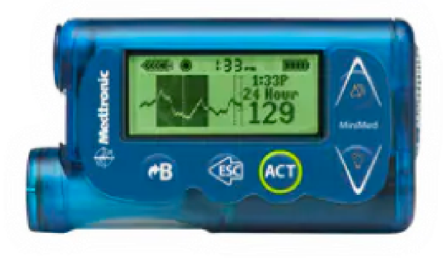


○

**InsulinType**

What type of insulin have you been using with your t:slim X2 insulin pump?

☐ Humalog (U-100) (Lispro)
☐ Humalog (U-200) (Lispro)
☐ Novolog (Aspart) (Can also be purchased under the name 'ReliOn')

- ☐ Apidra (Glulisine)
- ☐ Admelog
- ☐ Lantus, Basaglar, Semglee, or Toujeo (Glargine)
- ☐ FiAsp
- ☐ Lyumjev
- ☐ Levemir (Detemir)
- ☐ Tresiba (Degludec)
- ☐ Other (please specify): ______

**Diabetes Impact and Devices Satisfaction Scale**

The following questions ask about your experience in the last 3 months using.

How satisfied are you with?

| Very **Unsatisfied** | | | | | | | | | Very **Satisfied** |
|---|---|---|---|---|---|---|---|---|---|
| 1 ○ | 2 ○ | 3 ○ | 4 ○ | 5 ○ | 6 ○ | 7 ○ | 8 ○ | 9 ○ | 10 ○ |

How much do you trust?

| **Not at all** | | | | | | | | | **A lot** |
|---|---|---|---|---|---|---|---|---|---|
| 1 ○ | 2 ○ | 3 ○ | 4 ○ | 5 ○ | 6 ○ | 7 ○ | 8 ○ | 9 ○ | 10 ○ |

Please indicate how much you agree or disagree with each statement based on your experience using.

| | Strongly **Disagree** 1 | 2 | 3 | 4 | 5 | 6 | 7 | 8 | 9 | Strongly **Agree** 10 |
|---|---|---|---|---|---|---|---|---|---|---|
| ...is easy to use. | ○ | ○ | ○ | ○ | ○ | ○ | ○ | ○ | ○ | ○ |
| ...helps me have good blood glucose control. | ○ | ○ | ○ | ○ | ○ | ○ | ○ | ○ | ○ | ○ |
| ...is a hassle to use. | ○ | ○ | ○ | ○ | ○ | ○ | ○ | ○ | ○ | ○ |
| ...helps me feel more in control of my diabetes. | ○ | ○ | ○ | ○ | ○ | ○ | ○ | ○ | ○ | ○ |
| ...is too complicated. | ○ | ○ | ○ | ○ | ○ | ○ | ○ | ○ | ○ | ○ |

In the last 3 months, how often did you....

| | **Never** 1 | 2 | 3 | 4 | 5 | 6 | 7 | 8 | 9 | **Always** 10 |
|---|---|---|---|---|---|---|---|---|---|---|
| Have a bad night's sleep because of diabetes? | ○ | ○ | ○ | ○ | ○ | ○ | ○ | ○ | ○ | ○ |
| Wake up at night to treat low blood glucose? | ○ | ○ | ○ | ○ | ○ | ○ | ○ | ○ | ○ | ○ |
| Miss work, school, household chores, or other responsibilities due to diabetes? | ○ | ○ | ○ | ○ | ○ | ○ | ○ | ○ | ○ | ○ |
| Worry about going low? | ○ | ○ | ○ | ○ | ○ | ○ | ○ | ○ | ○ | ○ |

## Impact of Diabetes profile

**How does diabetes CURRENTLY impact the following aspects of your life?**
*Select one option on each line. Only use the N/A option if the statement is not applicable to you.*

| | Very negative impact | Negative impact | Slightly negative impact | No impact | Slightly positive impact | Positive impact | Very positive impact | N/A |
|---|---|---|---|---|---|---|---|---|
| Your physical health | ○ | ○ | ○ | ○ | ○ | ○ | ○ | ○ |
| Your financial situation | ○ | ○ | ○ | ○ | ○ | ○ | ○ | ○ |
| Your relationship with your family, friends and peers | ○ | ○ | ○ | ○ | ○ | ○ | ○ | ○ |
| Your leisure activities | ○ | ○ | ○ | ○ | ○ | ○ | ○ | ○ |
| Your work or studies | ○ | ○ | ○ | ○ | ○ | ○ | ○ | ○ |
| Your emotional well-being | ○ | ○ | ○ | ○ | ○ | ○ | ○ | ○ |
| Your freedom to eat as you wish | ○ | ○ | ○ | ○ | ○ | ○ | ○ | ○ |

## Sleep Question

The following question is about your quality of sleep.

Thinking about your sleep since you started using Control-IQ technology, how has your sleep quality improved?

| Very Poor | Poor | Average | Good | Very Good |
|---|---|---|---|---|
| ○ | ○ | ○ | ○ | ○ |

**AE Questions**

This section will include questions about any diabetes complications you have had in the **last month.**

First, we would like to ask you about severe hypoglycemia (low blood sugar) episodes.

> Definition of Severe Hypoglycemia:
> A severe hypoglycemia episode is a situation in which your blood sugar is low, and you need help from another person to help you raise your blood sugar by giving you sugar, carbs, glucagon or calling 911. The other person might be a friend or family member, or a healthcare professional.

**In the last month, did you have any severe hypoglycemia episodes that were treated with help from another person (e.g., friend, family member, medical/healthcare provider)?**

- ○ Yes
- ○ No

You indicated that you had severe hypoglycemia episode(s) in the last month. How many of them required a trip to the emergency room or hospital?

☐

These next few questions will ask about any episodes of diabetic ketoacidosis (DKA) you may have had in the last month.

Definition of diabetic ketoacidosis (DKA): DKA is a situation in which your blood sugar is high and each of the following occurred:

1. You felt sick to your stomach (with or without vomiting), and/or you were urinating more than usual and/or more thirsty than usual
2. You were treated in a health care facility
3. You were told by a health care provider that you had diabetic ketoacidosis or DKA

**In the last month, did you have any Diabetic Ketoacidosis (DKA) episodes, as defined above?**

○ Yes

○ No

You indicated that you had DKA episodes. How many required a trip to the emergency room or hospital?

## Demographics

In the last 3 months, what is the average number of insulin units you used per day? If you are unsure, please take your best guess.

Average # of Insulin Units Per Day:

When did you last have your HbA1c test done? If you are unsure, please take your best guess.

- In the past 2 weeks
- In the last month
- In the last 2 - 3 months
- In the last 4 - 6 months
- In the last 6 - 12 months
- Longer than 1 year ago

What was your most recent HbA1c value?  If you are unsure, please take your best guess.

What is your current height?

What is your current weight? (pounds)

What is your current relationship status?

- Never Married
- Married
- Prefer not to answer
- Living with a partner

- ○ Widowed
- ○ Divorced
- ○ Separated

On average, how often do you exercise in a week?

- ○ Once a week
- ○ 2-3 times per week
- ○ 4-5 times per week
- ○ 6+ times per week
- ○ I do not exercise regularly

Some people may experience other health complications while they are managing their diabetes. Please indicate if you are currently undergoing treatment or taking medications for any of the following.

- ☐ Eye problems
- ☐ Kidney problems
- ☐ Dental problems
- ☐ Numbness/tingling/loss of sensation in your feet
- ☐ High blood pressure
- ☐ High cholesterol
- ☐ Sexual problems
- ☐ Depression
- ☐ Anxiety
- ☐ None
- ☐ Prefer not to answer

Have you ever been diagnosed with Coronavirus (COVID-19)?

- ○ Yes
- ○ No
- ○ Prefer not to answer

## Open End Question

Is there anything else you would like to tell us about your experience with using? Please explain in the text box below.

## Withdraw

If you wish to withdraw from the study, please click here.

- ○ Withdraw from study
