# Supplementary material for: Real-World Use of Control-IQ Technology Is Associated with a Lower Rate of Severe Hypoglycemia and Diabetic Ketoacidosis Than Historical Data: Results of the Control-IQ Observational (CLIO) Prospective Study
Source: Diabetes Technol Ther. 2024 Jan 5;26(1):24–32. doi: 10.1089/dia.2023.0341 (PMC10794820; doi:10.1089/dia.2023.0341)
Supplement: Supplemental data [file Suppl_Data.zip › SupplementalMaterialSurveyMonthlyAE.pdf]

TANDEM® DIABETES CARE

CLIO Study: Month 1 Survey

**Inline Email**

In the first month using your t:slim X2 pump with Control-IQ technology, did you need help from another person for your low OR high blood sugar episodes because you were unable to treat them yourself?

- ○ Yes
- ○ No

**SH**

First, we would like to ask you about any severe hypoglycemia (low blood sugar) episodes you may have had in the last month during the following time frame:

Definition of Severe Hypoglycemia:

A severe hypoglycemia episode is a situation in which your blood sugar is low, and you need help from another person to help you raise your blood sugar by giving you sugar, carbs, glucagon or calling 911. The other person might be a friend or family member, or a healthcare professional.

**In the last month, did you have any severe hypoglycemia episodes that were treated with help from another person (e.g., friend, family member, medical/healthcare provider)?**

- ○ Yes
- ○ No

You indicated that you had severe hypoglycemia episode(s) in the last month. How many of them required a trip to the emergency room or hospital?

**DKA**

These next few questions will ask about any episodes of diabetic ketoacidosis (DKA) you may have had in the last month.

Definition of diabetic ketoacidosis (DKA): DKA is a situation in which your blood sugar is high and each of the following occurred:

1. You felt sick to your stomach (with or without vomiting), and/or you were urinating more than usual and/or more thirsty than usual
2. You were treated in a health care facility
3. You were told by a health care provider that you had diabetic ketoacidosis or DKA

**In the last month, did you have any Diabetic Ketoacidosis (DKA) episodes, as defined above?**

- ○ [ ] Yes
- ○ No

You indicated that you had DKA episodes. How many required a trip to the emergency room or hospital?

## Withdraw from study

If you wish to withdraw from the study, please click here.

○ Withdraw from study
