# Supplementary material for: Real-World Use of Control-IQ Technology Is Associated with a Lower Rate of Severe Hypoglycemia and Diabetic Ketoacidosis Than Historical Data: Results of the Control-IQ Observational (CLIO) Prospective Study
Source: Diabetes Technol Ther. 2024 Jan 5;26(1):24–32. doi: 10.1089/dia.2023.0341 (PMC10794820; doi:10.1089/dia.2023.0341)
Supplement: Supplemental data [file Suppl_TableS1.pdf]

Supplemental Table 1. Adverse Event Rates. Mean (SD) adverse event rates per 100 patient years for study participants as self-reported on monthly surveys through 12 months of Control-IQ technology use, compared to historical rates by prior baseline HbA1c, prior insulin delivery method (pump vs MDI), or prior CGM experience. The lower observed rate of AE's with Control-IQ technology use compared to historical data was independent of baseline HbA1c, prior insulin delivery method (pump vs MDI), or prior CGM experience, with all groups showing benefit. Effect size for t-test between groups was determined by Cohen's d. Significance was defined as p-value <0.05.

Adverse Event Rates	CIQ Rate	Historical Rate	Effect Size, p-value
**Baseline HbA1c < 8.0% (N=1,766)**			
Severe Hypoglycemia	9.66 (35.55)	24.02 (95.03)	d=0.40, p<0.01
Diabetic Ketoacidosis	0.96 (10.33)	2.60 (32.16)	d=0.16, p<0.01
**Baseline HbA1c ≥ 8.0% (N=1,295)**			
Severe Hypoglycemia	9.58 (37.03)	25.82 (98.28)	d=0.44, p<0.01
Diabetic Ketoacidosis	2.47 (16.50)	17.77 (82.40)	d=0.93, p<0.01
**Prior CGM Experience (N=2,669)**			
Severe Hypoglycemia	9.87 (36.99)	18.04 (83.01)	d=0.22, p<0.01
Diabetic Ketoacidosis	1.37 (12.02)	4.17 (40.65)	d=0.24, p<0.01
**No Prior CGM Experience (N=392)**			
Severe Hypoglycemia	7.99 (30.04)	27.69 (101.54)	d=0.66, p<0.01
Diabetic Ketoacidosis	3.57 (19.91)	13.23 (71.52)	d=0.48, p<0.01
**Prior Pump User (N=2,146)**			
Severe Hypoglycemia	9.61 (36.06)	20.77 (88.74)	d=0.31, p<0.01
Diabetic Ketoacidosis	1.26 (11.56)	7.75 (55.15)	d=0.56, p<0.01
**Prior MDI User (N=915)**			
Severe Hypoglycemia	9.65 (36.47)	35.58 (113.86)	d=0.71, p<0.01
Diabetic Ketoacidosis	2.40 (16.69)	17.79 (82.45)	d=0.92, p<0.01

CIQ = Control-IQ Technology
MDI = Multiple Daily Injections
