# Supplementary material for: Real-World Use of Control-IQ Technology Is Associated with a Lower Rate of Severe Hypoglycemia and Diabetic Ketoacidosis Than Historical Data: Results of the Control-IQ Observational (CLIO) Prospective Study
Source: Diabetes Technol Ther. 2024 Jan 5;26(1):24–32. doi: 10.1089/dia.2023.0341 (PMC10794820; doi:10.1089/dia.2023.0341)
Supplement: Supplemental data [file Suppl_TableS2.pdf]

Supplemental Table 2. Adverse Event Rates by Age Group. Mean (SD) adverse event rates per 100 patient years for study participants as self-reported on monthly surveys through 12 months of Control-IQ technology use, listed by age 6-13, 14-17, and age 18+.

Adverse Event Rates	CIQ Rate
**Age 18+ (N=2,130)**	
Severe Hypoglycemia	9.77 (36.97)
Diabetic Ketoacidosis	1.46 (13.10)
**Age 14-17 (N=412)**	
Severe Hypoglycemia	6.80 (26.15)
Diabetic Ketoacidosis	1.46 (11.99)
**Age 6-13 (N=519)**	
Severe Hypoglycemia	11.30 (39.53)
Diabetic Ketoacidosis	2.31 (15.04)

CIQ = Control-IQ Technology
