# Supplementary material for: Real-World Use of Control-IQ Technology Is Associated with a Lower Rate of Severe Hypoglycemia and Diabetic Ketoacidosis Than Historical Data: Results of the Control-IQ Observational (CLIO) Prospective Study
Source: Diabetes Technol Ther. 2024 Jan 5;26(1):24–32. doi: 10.1089/dia.2023.0341 (PMC10794820; doi:10.1089/dia.2023.0341)
Supplement: Supplemental data [file Suppl_TableS3.pdf]

Supplemental Table 3. Safety analysis of the auto-populated CGM value into the bolus calculator with resultant hypoglycemia. The percentage of boluses using the auto-population feature (where CGM values auto-filled into the bolus calculator) result in fewer readings <54 mg/dL and <70 mg/dL than those not using the feature (where glucose values were manually entered into the bolus calculator to override the autopopulation of CGM values), in every pre-bolus glucose range examined. There is no evidence of increased risk of hypoglycemia when auto-populated CGM results are used to calculate the subsequent bolus.

Glucose range prior to bolus	CGM auto-populated?	Total (count)	At least one reading < 54 mg/dL (count)	At least one reading < 54 mg/dL (%)	At least one reading < 70 mg/dL (count)	At least one reading < 70 mg/dL (%)	At least 5 readings < 70 mg/dL (count)	At least 5 readings < 70 mg/dL (%)	At least 3 consecutive readings < 70 mg/dL (count)	At least 3 consecutive readings < 70 mg/dL (%)
**70 to 180 mg/dL Correction Bolus Results within 5 hours after bolus**	Yes	1,765,869	68,782	3.9	232,769	13.18	119,445	6.76	166,386	9.42
**70 to 180 mg/dL Correction Bolus Results within 5 hours after bolus**	No	59,228	2,528	4.27	8,601	14.52	4,356	7.35	5,954	10.05
**181 to 250 mg/dL Correction Bolus Results within 5 hours after bolus**	Yes	977,919	33,802	3.46	108,167	11.06	54,531	5.58	75,992	7.77
**181 to 250 mg/dL Correction Bolus Results within 5 hours after bolus**	No	14,235	823	5.78	2,222	15.61	1,188	8.35	1,560	10.96
**> 250 mg/dL Correction Bolus Results within 5 hours after bolus**	Yes	302,078	10,984	3.64	31,239	10.34	16,012	5.3	22,451	7.43
**> 250 mg/dL Correction Bolus Results within 5 hours after bolus**	No	8,799	500	5.68	1188	13.5	631	7.17	834	9.48
**All**	Yes	3,045,888	113,571	3.73	372,188	12.22	189,992	6.24	264,837	8.69
**All**	No	82,262	3,851	4.68	12,011	14.6	6,175	7.51	8,348	10.15
