# Supplementary material for: Real-World Use of Control-IQ Technology Is Associated with a Lower Rate of Severe Hypoglycemia and Diabetic Ketoacidosis Than Historical Data: Results of the Control-IQ Observational (CLIO) Prospective Study
Source: Diabetes Technol Ther. 2024 Jan 5;26(1):24–32. doi: 10.1089/dia.2023.0341 (PMC10794820; doi:10.1089/dia.2023.0341)
Supplement: Supplemental data [file Suppl_TableS4.pdf]

Supplemental Table 4. Number of participant surveys completed. Monthly surveys were not sent out until after a data upload showed first evidence of closed-loop use. Since surveys had to be completed within 30 days of the end of each month, for a number of participants this was after their window to complete the first few surveys.

Survey	Surveys Partially Completed	Surveys Fully Completed	Full Completion Rate (%) of Sent Surveys
Baseline Survey	5,532	5,009	90.5
Month 1 AE Survey	1,824	1,802	88.2
Month 2 AE Survey	2,547	2,507	90.0
Month 3 AE and PRO Survey	2,633	2,605	90.9
Month 4 AE Survey	2,717	2,689	90.8
Month 5 AE Survey	2,740	2,704	96.7
Month 6 AE and PRO Survey	2,734	2,700	89.0
Month 7 AE Survey	2,635	2,594	92.7
Month 8 AE Survey	2,704	2,695	88.8
Month 9 AE Survey	2,801	2,776	89.1
Month 10 AE Survey	2,784	2,770	89.4
Month 11 AE Survey	3,133	2,756	93.2
Month 12 AE and PRO Survey	2,818	2,778	90.5
